# Anti-Inflammatory and Antiarthritic Activity of Anthraquinone Derivatives in Rodents

**DOI:** 10.1155/2014/690596

**Published:** 2014-12-24

**Authors:** Ajay D. Kshirsagar, Prashant V. Panchal, Uday N. Harle, Rabindra K. Nanda, Haidarali M. Shaikh

**Affiliations:** ^1^School of Pharmacy, Swami Ramanand Teerth Marathwada University, Vishnupuri, Nanded, Maharashtra 431606, India; ^2^Pad. Dr. D. Y. Patil Institute of Pharmaceutical Sciences and Research, Pimpri, Pune, Maharashtra 411018, India

## Abstract

Aloe emodin is isolated compound of aloe vera which is used traditionally as an anti-inflammatory agent. In vitro pharmacokinetic data suggest that glucuronosyl or sulfated forms of aloe emodin may provide some limitations in its absorption capacity. Aloe emodin was reported to have in vitro anti-inflammatory activity due to inhibition of inducible nitric oxide (iNO) and prostaglandin E_2_, via its action on murine macrophages. However, present work evidenced that molecular docking of aloe emodin modulates the anti-inflammatory activity, as well as expression of COX-2 (cyclooxygenase-2) in rodent. The AEC (4,5-dihydroxy-9,10-dioxo-9,10-dihydroanthracene-2 carboxylic acid) was synthesized using aloe emodin as starting material. The study was planned for evaluation of possible anti-inflammatory and antiarthritic activity in carrageenan rat induced paw oedema and complete Freund's adjuvant induced arthritis in rats. The AE (aloe emodin) and AEC significantly (*P* < 0.001) reduced carrageenan induced paw edema at 50 and 75 mg/kg. Complete Freund's adjuvant induced arthritis model showed significant (*P* < 0.001) decrease in injected and noninjected paw volume, arthritic score. AE and AEC showed significant effect on various biochemical, antioxidant, and hematological parameters. Diclofenac sodium 10 mg/kg showed significant (*P* < 0.001) inhibition in inflammation and arthritis.

## 1. Introduction

Rheumatoid arthritis (RA) is a chronic systemic autoimmune disease characterized by nonspecific inflammation of peripheral joints, destruction of articular tissues, and deformities in the joints. As the disease progresses, there are enhanced chances of bone damage and destruction of cartilage causing substantial disability [1]. The consequent morbidity and mortality have a substantial socioeconomic impact. The pathological conditions of RA are well known such as the leukocyte infiltration, a chronic inflammation, pannus formation, and extensive destruction of the articular cartilage and bone. The exact cause of RA is not yet known.

In particular, it was reported that the inflammatory cytokines, such as tumor necrosis factor- (TNF-) *α*, interleukin- (IL-) 1*β*, and IL-6, play key roles in the inflammation and joint damages during the development of RA [2]. Epidemiology of the arthritis in female to male is 3 : 1 and the prevalence is 1% of the world population [3]. Nonsteroidal anti-inflammatory drugs (NSAIDs) are widely used for the treatment of rheumatism diseases, such as rheumatoid arthritis and pain. The pharmacological effects of NSAIDs are due to inhibition of a membrane enzyme called cyclooxygenase (COX) which is involved in the prostaglandin biosynthesis [4].

In spite of their extensive usage, NSAIDs are associated with many adverse effects like myocardial infarction: Rofecoxib [5], gastric irritation (Indomethacin), loose stool, nausea, vomiting, and dyspepsia (ketorolac). Chronic usage of Aspirin may also lead to gastric ulceration and liver damage [6, 7]. Nonsteroidal anti-inflammatory drugs are widely used in the treatment of a number of inflammatory conditions, but gastrointestinal (GI) lesions have often limited their clinical utilization. Topically applied NSAIDs rarely exhibit systemic side effects and most of the side effects are dermatological in nature-like rashes and/or pruritis. Adverse effects of the NSAIDs are usually dose related [8]. The fact of NSAIDs associated gastric damage is well explored for involvement of COX related pathway [9].

The naturally occurring 1,8-dihydroxyanthraquinone derivatives (1,8 DAD) were obtained from various families such as Rhamnaceae (buckthorn, cascara), Liliaceae (aloe), Polygonaceae (rhubarbs), and Caesalpiniaceae (senna) [10]. Aloe emodin is an anthraquinone glycoside having antioxidant [11], neuroprotective, and in vitro anti-inflammatory activity due to inhibition of inducible nitric oxide (iNO) and prostaglandin E_2_, via its action on murine macrophages [12].

Hence, taking into consideration reported activity of aloe emodin and molecular docking methodology, anthraquinone derivative was synthesized. The objective of forming this derivative was an attempt to reach an active anti-inflammatory agent with potent activity and selectivity toward COX-2. Molecular docking studies were carried out on these compounds to identify the structured feature required for effective bind to COX-2 enzyme. The most effectively bound ligand was taken as the active compound.

Hence, the present study was planned to explore possible modulation of pro-anti-inflammatory potential by modifying the aloe emodin into its derivative for evaluation of anti-inflammatory potential by considering the docking results, because no such study has been carried out in the past.

## 2. Materials and Methods

### 2.1. Materials

The aloe emodin was procured from aloe vera synthesis, Mumbai (India). Carrageenan was purchased from S. D. Fine lab. (India), Diclofenac was obtained from Hindustan Antibiotics Ltd. (India), and complete Freund's adjuvant was obtained from Sigma-Aldrich (USA).

### 2.2. Docking Study

The coordinates for the three-dimensional structure of COX-2 were obtained from Protein Data Bank (http://www.rcsb.org/pdb/, entry code 4COX) [13, 14]. Water molecules were removed and hydrogens were added to the structure of protein before docking. The structures of ligands (AE and AEC) selected for the docking study were constructed using standard bond lengths and angles using Advanced Chemistry Development (ACD) ChemSketch software. As the cocrystal structure of 4COX showed eight cavities, the selection of cavity for docking studies of the synthesized ligands was made on the basis of initial docking study. The docking study of aloe emodin and its derivative was carried out using VLife MDS docking software 3.0. The binding energy of ligands to the 4COX was calculated by using the following formula:

 
*G*_binding_ = *G*_complex_ − (*G*Δ_ligand_ + *G*_protein_),
 
*G*_complex_ = *G*(ligand + protein),
 
*G*_protein_ = the energy of the protein after optimization (uncomplexed),
 
*G*_ligand_ = the energy of the ligand after optimization (uncomplexed),
 Δ*G*_binding_ is binding energy required for a ligand to bind a protein.

### 2.3. Chemical Study

**Scheme 1:**
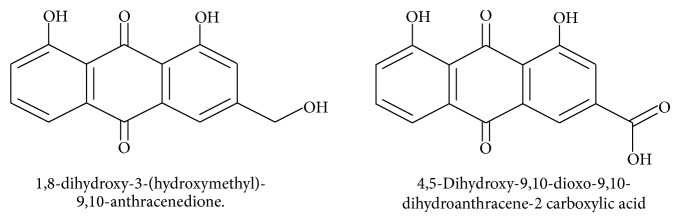
Structures of aloe emodin and its carboxylic acid derivative.

*Synthesis of 4,5-Dihydroxy-9,10-dioxo-9,10-dihydroanthracene-2 Carboxylic Acid from Aloe Emodin [15] (See Scheme 1)*

**Scheme 2:**
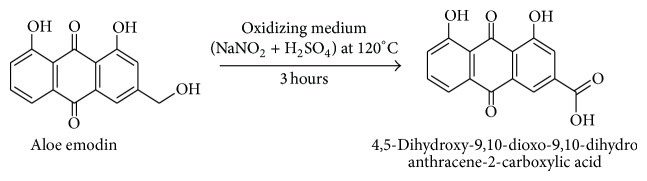


*Reaction.* See Scheme 2.

*Procedure.* In this procedure the oxidizing medium was prepared by dissolving 2.5 g of sodium nitrite in 12 mL of sulphuric acid; the solution was heated to about 120°C. One gram of aloe emodin was added in parts to this mixture over a period of 30 min. The reaction mixture was kept at this temperature for 3 h. At the end of 3 h the reaction mixture was poured into 700 mL distilled water at 2°C to get orange brown precipitate (containing a mixture of 4,5-dihydroxy-9,10-dioxo-9,10-dihydroanthracene-2 carboxylic acid and the starting material aloe emodin). The precipitate so formed was filtered and dried to obtain crude 4,5-dihydroxy-9,10-dioxo-9,10-dihydroanthracene-2 carboxylic acid. This was then dissolved in sodium carbonate solution pH below 9.5 and extracted with organic solvent. The unreacted aloe emodin present gets extracted into the organic solvent (Dichloromethane). The compound is again regenerated from sodium bicarbonate solution using hydrochloric acid. The precipitate is then filtered, washed, dried, and recrystallized from methanol to obtain pure compound with yield of 95%.

### 2.4. Experimental Animals

*Rats.* Albino Wistar rats of either sex weighing 150–250 g were used for present study. They were kept in polypropylene cages in an air-conditioned area at 22 ± 3°C in 10–14 h light dark cycle. They were provided with balanced feed and water* ad libitum*.

The experimental protocol was approved by IAEC (Institutional Animal Ethics Committee). Laboratory animal handling and experimental procedures were performed in accordance with CPCSEA guidelines (Approval number: 198/99).

### 2.5. Pharmacological Studies

*Carrageenan Induced Paw Edema in Rats.* Anti-inflammatory activity of AE and AEC was tested using the carrageenan induced rat paw edema model [16]. Experimental animals (Wistar rats) were randomly divided into eight groups with six animals in each group. Group I (control group) received vehicle (1% CMC). Group II (standard group) received Diclofenac sodium at dose 10 mg/kg. Groups III–V (AE) received aloe emodin at dose of 25, 50, and 75 mg/kg, respectively. Groups VI–VIII (AEC) received aloe emodin derivative at dose of 25, 50, and 75 mg/kg. The drugs were administered orally 1 h prior to the injection of 0.1 mL of freshly prepared suspension of carrageenan into the left hind paw of each rat. The paw volume was measured using a Plethysmometer (Ugo Basile 7140, Italy) at the time interval of 0.5 hr, 1 hr, 2 hr, 3 hr, 4 hr, 5 hr, and 24 hr after administration of carrageenan. Results were expressed as

(1) $$\begin{array}{c} \text{Edema}\;\text{volume}=V_{t}-V_{c}, \end{array}$$

where *V*_*t*_ is paw volume in mL, at time *t*, after carrageenan administration. *V*_*c*_ is paw volume in mL, before carrageenan administration. Consider

(2) $$\begin{array}{c} \text{Inhibition}\;\text{rate}\;\;\left(\%\right)=\frac{{E_{c}-E}_{t}}{E_{c}}\times 100, \end{array}$$

where *E*_*c*_ is edema volume of control group. *E*_*t*_ is edema volume of treated group.

*Complete Freund's Adjuvant Induced Arthritis in Rats.* Adjuvant arthritis was induced as previously described by [17] as modified by [18]. On day 0, for the induction of arthritis, all the animals were anesthetized with intraperitoneal injections of 40 mg/kg thiopentone (0.3 mL/300 g rat) and arthritis was induced by the injection of 0.1 mL of complete Freund's adjuvant (CFA) containing 1.0 mg dry heat-killed* Mycobacterium tuberculosis* per milliliter sterile paraffin oil into tibiotarsal joint of the left hind paw of female rats. The female Wistar rats weighing 180–230 g were divided into eight groups of six animals in each group as follows:

 group I: arthritic control/CFA (intraplantar injection of 0.1 mL CFA);
 group II: standard treated with Diclofenac sodium 10 mg/kg after intraplantar injection of 0.1 mL CFA, from 12th to 28th day;
 groups III–V: treated with AE 25, 50, and 75 mg/kg, respectively, after intraplantar injection of 0.1 mL CFA, from 12th to 28th day;
 groups VI–VIII: treated with AEC 25, 50, and 75 mg/kg, respectively, after intraplantar injection of 0.1 mL CFA, from 12th to 28th day.

The following parameters were measured.

*(A) Paw Volume Evaluation (in mL).* Paw volume was measured on days 0, 4, 8, 14, 21, and 28 by using Plethysmometer (UGO Basile, 7140, Italy). Mean changes in injected and noninjected paw edema, with respect to initial paw volume, were calculated on respective day and % inhibition of paw edema with respect to untreated group was calculated using the following formula:

(3) $$\begin{array}{c} i=\left\{1-\left(\frac{\Delta v\;\;\text{treated}}{\Delta v\;\;\text{Untreated}}\right)\right\}\times 100, \end{array}$$

where *i* is % inhibition of paw edema and Δ*v* treated is mean changes in paw volume of treated rat. Δ*v* untreated is mean changes in paw volume of untreated rat.

*(B) Visual Arthritis Scoring System.* The visual arthritis scoring system described by [19, 20] was used to evaluate the severity of arthritis. In this scoring system each paw of animal was observed and separate score was given for each limb. Observations are recorded by observer who was blind to the study. The arthritis score ranged from 0 to 4, where 0 indicated the least but definite swelling and 4 represented the maximum swelling.

*(C) Antioxidants.* On the 28th day, animals were anaesthetized by ether and sacrificed by cervical dislocation. The liver of each animal was removed rapidly and washed with ice cold Tris buffer. Liver of each animal was cut into small pieces and homogenized with homogenizer, so that clear homogenate is formed. Homogenates were used for estimation of LPO (lipid hydroperoxide), GSH, SOD, and catalase. Reduced glutathione (GSH) in liver was estimated according to the method described by [21]. The concentration of reduced glutathione was expressed as *μ*g of GSH/g of tissue. Superoxide dismutase (SOD) activity was estimated using the technique of [22]. The SOD activity (units/mg of tissue) was calculated by using the standard plot. The catalase (CAT) activity was determined according to the method of [23, 24]. CAT activity was expressed as *μ*mol H_2_O_2_ decomposed/min/g of tissue. The LPO (lipid hydroperoxide) end product malondialdehyde (MDA) measured in the homogenate was estimated by using method of [25]. The concentration of lipid peroxidation was expressed as nmol/g of MDA/g of tissue.

*(D) Nitrite Content.* The accumulation of nitrite in the supernatant, an indicator of the production of nitric oxide (NO), was determined with a colorimetric assay with Griess reagent (0.1% N-(1-Naphthyl)ethylenediamine dihydrochloride, 1% sulfanilamide, and 2.5% phosphoric acid). The concentration of nitrite *i* was determined from a sodium nitrite standard curve.

*(E) Hematological Parameters.* On the 28th day, blood was withdrawn through retroorbital plexus puncture from all groups by under light ether anesthesia and the hematological parameters like hemoglobin content, total WBC count, ESR, and RBC were analyzed using Culter CB-9000, Chariot.

*(F) Biochemical Parameter.* On the 28th day, blood was withdrawn through retroorbital plexus of all groups and the biochemical parameters SGPT (serum glutamate pyruvic transaminase), SGOT (serum glutamic oxaloacetic transaminase), and ALP were analyzed using standard kits followed by fully automated analyzer.

### 2.6. Statistical Analysis

Values were expressed as mean ± SEM (*n* = 6). Statistical significance was determined using one-way ANOVA followed by Dunnett test. ^∗^ *P* < 0.05, ^∗∗^ *P* < 0.01 when compared with control group.

## 3. Result

### 3.1. Docking Studies

**Figure 1:**
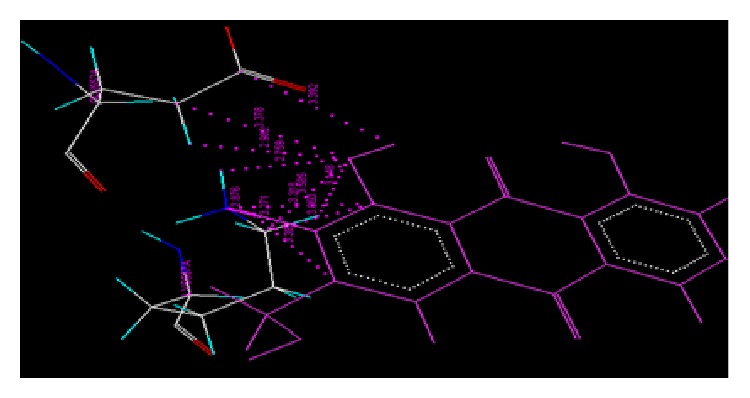
Van der Waals interaction of AE with protein 4COX.

**Figure 2:**
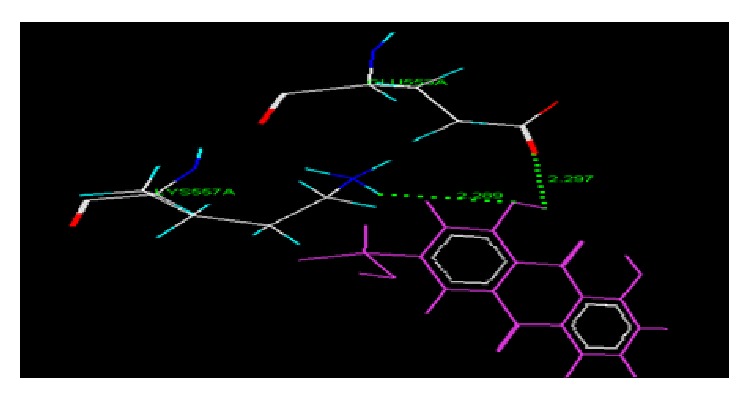
Hydrogen bond interaction of AE with protein 4COX.

**Figure 3:**
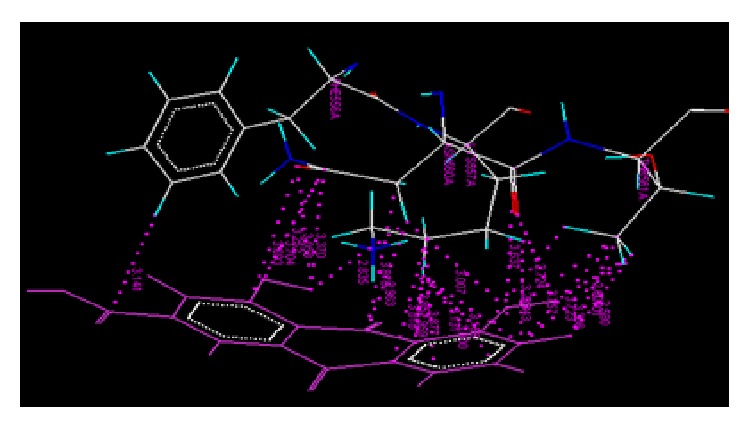
Van der Waals interaction of AEC with protein 4COX.

The results of docking studies of anthraquinone derivatives (Dock score and net binding energy Δ*G*) on cyclooxygenase protein (4COX) using VLife MDS were as shown in Table 1. The interactions between the active amino acid residues of the protein and ligand molecules are enlisted and diagrammatically represented. Figures 1 and 2 represent the Van der Waals and hydrogen bond interaction, respectively, of AE with 4COX protein. The amino acid involved, type of interaction, and ligand atom involved in interaction are shown in Table 2.  Figure 3 represents the Van der Waals interaction of AEC with 4COX protein. The amino acid involved, type of interaction, and ligand atom involved in interaction are shown in Table 3.

From the docking study it was observed that the common amino acids which interact with the common ligand were GLU 553A, LYS557A, ASN560A, and THR561A. The compound AEC was most active while compound AE was least active as per docking.

### 3.2. Carrageenan Induced Rat Paw Edema

In the carrageenan induced rat paw edema model of anti-inflammatory activity, the AE and AEC showed a significant inhibitory effect of the edema formation from the first hour to the sixth hour and after twenty-four hours. The highest inhibitory effect was found in late phase, that is, after the third hour (*P* < 0.01) at doses of 50 and 75 mg/kg when compared with control group (Table 4).

### 3.3. Complete Freund's Adjuvant Induced Arthritis in Rats

**Figure 4:**
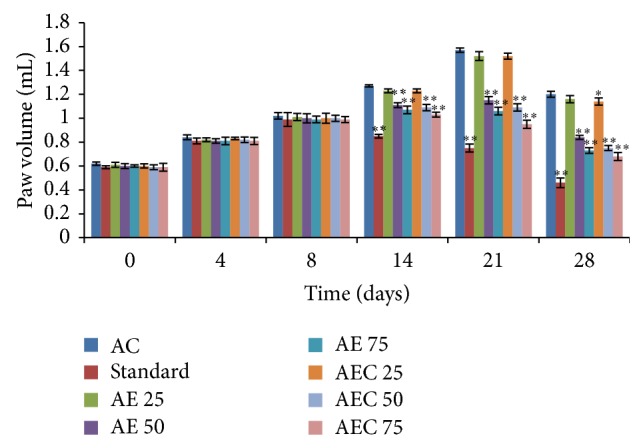
Effect of AE and AEC on injected paw volume in Freund's complete adjuvant induced arthritic rats. Results were presented as mean ± SEM (*n* = 6). The data was analysed using one-way analysis of variance (ANOVA) followed by Dunnett test. ^∗^ *P* < 0.05, ^∗∗^ *P* < 0.01 when compared with arthritic control group. AC: arthritic control; Std.: Diclofenac sodium 10 mg/kg p.o.; AE: aloe emodin (25, 50, and 75 mg/kg p.o.); AEC: carboxylic acid derivative of aloe emodin (25, 50, and 75 mg/kg p.o.).

**Figure 5:**
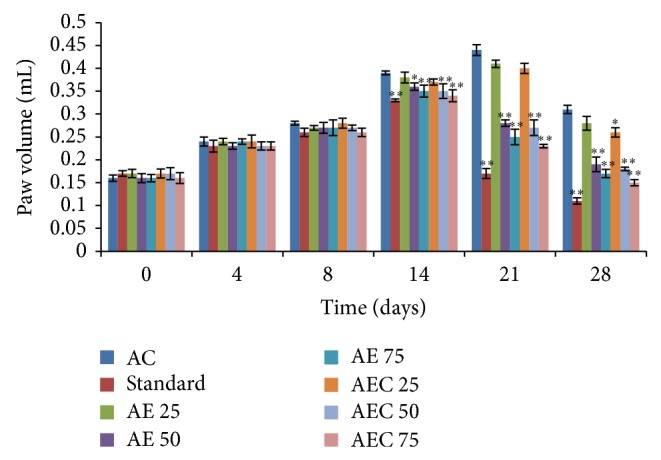
Effect of AE and AEC on noninjected paw volume in Freund's complete adjuvant induced arthritic rats. Results were presented as mean ± SEM (*n* = 6). The data was analysed using one-way analysis of variance (ANOVA) followed by Dunnett test. ^∗^ *P* < 0.05, ^∗∗^ *P* < 0.01 when compared with arthritic control group. AC: arthritic control; Std.: Diclofenac sodium 10 mg/kg p.o.; AE: aloe emodin (25, 50, and 75 mg/kg p.o.); AEC: carboxylic acid derivative of aloe emodin (25, 50, and 75 mg/kg p.o.).

Treatment with Diclofenac sodium (10 mg/kg), AE (50 and 75 mg/kg), and AEC (50 and 75 mg/kg) showed significant decrease in injected paw edema volume on the 14th, 21st, and 28th day (*P* < 0.001) as compared to arthritic control group. Group treated with AEC 25 showed significant decrease in paw volume on the 28th day (*P* < 0.05) as compared to arthritic control (Figure 4). Groups treated with Diclofenac sodium, AE (75 mg/kg), and AEC (50 and 75 mg/kg) showed significant decrease in the noninjected paw edema volume on the 14th, 21st, and 28th day (*P* < 0.01) as compared to arthritic control. AE 50 treated groups showed significant decrease in the noninjected paw edema volume on the 14th day (*P* < 0.05) also on the 21st and 28th day (*P* < 0.01) as compared to arthritic control. AEC 25 treated groups showed significant decrease in the noninjected paw edema volume on the 28th day (*P* < 0.05) as compared to arthritic control (Figure 5).

**Figure 6:**
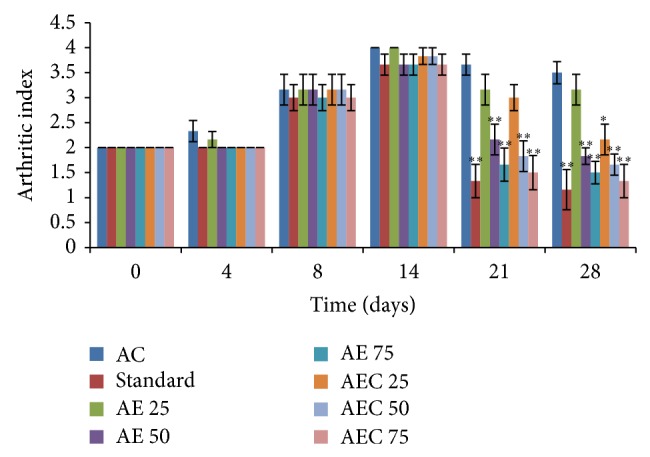
Effect of AE and AEC on arthritic index in Freund's complete adjuvant induced arthritic rats. Results were presented as mean ± SEM (*n* = 6). The data was analysed using one-way analysis of variance (ANOVA) followed by Dunnett test.^∗^ *P* < 0.05, ^∗∗^ *P* < 0.01 when compared with arthritic control group. AC: arthritic control; Std.**:** Diclofenac sodium 10 mg/kg p.o.; AE: aloe emodin (25, 50, and 75 mg/kg p.o.); AEC: carboxylic acid derivative of aloe emodin (25, 50, and 75 mg/kg p.o.).

Arthritic control group treated with Freund's complete adjuvant showed increase in the arthritic index from the 4th day up to the 28th day, respectively, while Diclofenac sodium treated group showed significant decrease in the arthritic index on the 14th, 21st, and 28th day (*P* < 0.01) as compared to arthritic control. Groups treated with AE 50, AE 75, AEC 50, and AEC 75 showed significant decrease in the arthritic index, on the 21st and 28th (*P* < 0.001) day, respectively, while AEC 25 treated groups showed significant decrease in the arthritic index on the 28th day (*P* < 0.05) as compared to arthritic control (Figure 6).

There was rise in WBC count and decrease in RBC and Hb count in arthritic control group. Diclofenac sodium treated group showed significant decrease in WBC count, rheumatoid factor (RF), and erythrocyte sedimentation rate (ESR) (*P* < 0.01), while it showed significant increase in RBC and Hb count (*P* < 0.01), as compared to arthritic control. Groups treated with AE 50, AE 75, AEC 50, and AEC 75 showed significant decrease in WBC count, RF, and ESR (*P* < 0.01), while they showed significant increase in RBC and Hb count (*P* < 0.01), as compared to arthritic control. Group treated with AEC 25 showed significant decrease in WBC count and RF (*P* < 0.05) when compared with arthritic control (Tables 5 and 6).

There was an increase in LPO and NO level and decrease in GSH, CAT, and SOD level in arthritic control group. Diclofenac sodium treated group showed significant decrease in the LPO and NO level (*P* < 0.01) as compared to arthritic control and significant increase in GSH, CAT, and SOD (*P* < 0.01) as compared to arthritic control. Groups treated with AE 50, AE 75, AEC 50, and AEC 75 showed significant increase in GSH, CAT, and SOD (*P* < 0.01) as compared to arthritic control. Group treated with AEC 25 showed significant decrease in LPO and NO level (*P* < 0.05), while it showed significant increase in GSH, CAT, and SOD (*P* < 0.05) as compared to arthritic control (Table 7).

Diclofenac sodium treated group showed significant decrease in SGPT, SGOT, and ALP levels (*P* < 0.01) as compared to arthritic control. Groups treated with AE 50, AE 75, AEC 50, and AEC 75 showed significant decrease in SGPT, SGOT, and ALP levels (*P* < 0.01) as compared to arthritic control. Group treated with AEC 25 showed significant decrease in SGOT level (*P* < 0.05) as compared to arthritic control (Table 7).

## 4. Discussion

Rheumatoid arthritis (RA) is a symmetric polyarticular arthritis that primarily affects the small diarthrodial joints of the hands and feet. In addition to inflammation in the synovium, which is the joint lining, the aggressive front of tissue called pannus invades and destroys local articular structures. The synovium is normally a relatively a cellular structure with a delicate intimal lining. In RA, CD4+ T cells, B cells, and macrophages infiltrate the synovium and sometimes organize into discrete lymphoid aggregates with germinal centres. Hyperplasia of the intimal lining results from a marked increase in macrophage-like and fibroblast-like synoviocytes. Locally expressed degradative enzymes, including metalloproteinases, serine proteases, and aggrecanases, digest the extracellular matrix and destroy the articular structures [26].

The structure of COX-2 was obtained from Protein Data Bank (PDB Code: 4COX) and used as target for docking studies. The docking study was carried out on VLife MDS docking software. The docking study was carried out on aloe emodin (AE) and carboxylic acid derivative of aloe emodin (AEC). The docking results of AEC were compared with that of AE based on various parameters such as Dock score, bond interactions, and binding free energy. The docking result showed that the AEC have favorable interactions with active site residues and also have favorable Dock score and binding free energy as compared with aloe emodin (AE), indicating that AEC may be having a better anti-inflammatory activity than aloe emodin.

In the present study, AE and AEC exhibited significant anti-inflammatory and antiarthritic activity. The carrageenan is known for its classic biphasic effect; the first phase is mediated by release of histamine and serotonin during the first hour and release of kinins up to 2.5 h, while the second phase is mediated by release of prostaglandins from 2.5 to 6 h [27]. It has been reported that the second phase is found to be sensitive to most of the clinically effective anti-inflammatory drugs [16, 28]. Hence, carrageenan induced inflammation is a nonspecific inflammation resulting from diverse mediators. This model is sensitive, conventional, and accepted for screening of newer anti-inflammatory agents. In the present study, AE and AEC showed dose-dependent inhibition of second phase of carrageenan induced rat paw edema, suggesting the inhibition of prostaglandins release.

In the present study, rats were selected to induce arthritis because rats develop a chronic swelling in multiple joints with influence of inflammatory cells, erosion of joint cartilage, and bone destruction. It has close similarities to human rheumatoid disease [29]. The pathogenesis or reasons for development of arthritis following injection of complete Freund's adjuvant preparations include reactivity to cartilage proteoglycans, heat shock proteins, and interactions with intestinal flora [30, 31]. The animals on exposure to CFA (or mycobacteria) in the early phases induce the release of cytokines such as TNF-*α*, IL-12, IL-6, and IFN-*γ* and several chemokines [32].

Paw swelling is an index of measuring the antiarthritic activity of various drugs [33]. The determination of paw swelling is simple, sensitive, and quick procedure for evaluating and assessing the degree of inflammation and the therapeutic and curative effects of drugs [34]. There is edema of periarticular tissues such as ligaments and joint capsules. The swelling increases in the initial phase of inflammation and then becomes constant in two weeks. These changes in paw volume are associated with increase in granulocytes and monocytes [35]. In chronic inflammation activation of macrophages results in the production of several cytokines including IL-1, IL-6, interferon-*γ*, and TNF-*α* which have been implicated in immune arthritis [36, 37]. IL-6 is considered to play a central role in chronic inflammation and is expressed in excess at sites of inflammation. Like IL-1 and TNF, IL-6 stimulates acute phase protein production. It also elicits the development of specific cellular and humoral immune responses such as B cell differentiation and T cell activation [38]. TNF-*α* is mainly involved in the perpetuation of the inflammatory cascades in autoimmune diseases, which affect connective tissues where the connective tissues become hypercontracted due to inflammation [39].

Prostaglandins greatly potentiate exudates by inducing relaxation of arteriolar smooth muscle cells, increasing the blood supply to the tissue [40]. In the present study, the standard drug Diclofenac sodium and test drugs AE and AEC significantly suppressed the paw edema swelling induced by the complete Freund's adjuvant (CFA), around tibiotarsal joint and paws. This indicates the anti-inflammatory activity of AE and AEC in rheumatoid arthritis.

The appearance of secondary lesions, that is, CFA-noninjected paw swelling, is a manifestation of cell-mediated immunity. The appearance of secondary lesions is due to development of biochemical reactions into CFA-noninjected hind leg which results in swelling around tibiotarsal joint and paw. The suppression of such secondary lesions by a drug shows its immunosuppressive activity [41, 42]. The AE and AEC effectively reduced the secondary lesions in arthritic rats. This reveals potent suppression by AE and AEC of cell-mediated immunity in arthritic rats. A selective reduction in the secondary lesions distinguishes the immunosuppressive effects of a drug from its anti-inflammatory effects. The significant reduction of the secondary lesions by AE and AEC as observed in this study indicates a possible immunosuppressant effect.

Anemia is commonly noted in patients with chronic arthritis [43]. The two most common explanations are gastrointestinal blood loss due to arthritis medications and bone marrow changes in patients with inflammatory arthritis, which prevents the release of iron for incorporation into red blood cells [44, 45]. In CFA-induced arthritis model, arthritic control rats showed reduced RBC count, reduced Hb count, and increased erythrocyte sedimentation rate (ESR) and RF levels. It is proposed that the reduction in the Hb count during arthritis results from reduced erythropoietin levels, a decreased response of the bone marrow erythropoietin, and premature destruction of red blood cells. Similarly, an increase in the ESR is attributed to the accelerated formation of endogenous proteins such as fibrinogen and *α*/*β* globulin, and such a rise in the ESR indicates an active but obscure disease process [46]. The acute phase proteins in ESR share the property of showing elevations in the concentration in response to stress or inflammations like injection, injury, surgery, and tissue necrosis [47].

Prominent immunologic abnormalities that may be important in pathogenesis of RA include immune complexes that are found in joint fluid cells and in vasculitis. Plasma cells produce antibodies (e.g., IgM) that contribute to these complexes. Serum RF measures the amount of antibody IgM titre present in the serum [48]. RF is the immunological expression of an individual's immune system reaction to the presence of an immunoglobulin molecule that is recognized as nonself. This response to the nonself immunoglobulin results in the presence of immune complexes; these in turn bind to the complement and may eventually lead to destruction of synovium, cartilage, and bone. The higher the levels of serum RF are, the higher the development of inflammation is [49]. Determination of serum RF levels in rheumatoid arthritis is essential to understand and measure the disease progression and to facilitate the development of novel treatments for rheumatoid arthritis. Serum RF is a marker of systemic inflammation and antibody production against the injected adjuvant. In CFA-induced arthritic rats, activated CD4+ T cells stimulate B cells to produce immunoglobulins, which are associated with increase in the plasma levels of serum RF [50, 51]. The AE and AEC treated groups showed a significant recovery from the induced anemia and serum RF level. This indicates that anemic conditions occurring during the inflammation in rheumatoid arthritis can be recovered by the treatment of AE and AEC.

In arthritic condition there is a mild to moderate rise in WBC count due to release of IL-Ib inflammatory response. IL-Ib increases the production of both granulocyte and macrophages colony stimulating factor [52, 53]. In the present study, the migration of leucocytes into the inflamed area was significantly suppressed by the standard drug, AE and AEC.

The body has effective antioxidant mechanism to prevent and neutralize the free radical induced damage. This is accomplished by a set of endogenous antioxidant enzymes, such as SOD and CAT. When the balance between ROS (reactive oxygen species) production and antioxidant defense is lost, “oxidative stress” results, which through a series of events deregulates the cellular function leading to various pathological conditions [54].

Biological systems have evolved an array of enzymatic and nonenzymatic antioxidant defense mechanisms to combat the deleterious effects of oxidative free radicals (OFRs). SOD is a metalloprotein while CAT is a hemoprotein, localized in the peroxisomes or the microperoxisomes. Both superoxide dismutase (SOD) and catalase play an important role in the detoxification of superoxide anion and H_2_O_2_, respectively, thereby protecting the cells against oxidative free radicals induced damage. H_2_O_2_ may be reduced by enzymes glutathione peroxide but, alternatively, may react again with superoxide anion to form free hydroxyl radicals, which have a greater toxicity and a longer half-life than superoxide anion. Although catalase is significantly increased in rheumatoid arthritis its concentration is very low to expect considerable protection against H_2_O_2_ [55, 56]. In arthritis the lowered levels of SOD activity may be due to the inhibition of the enzyme by hydrogen peroxide, which might be an indicator of high degree of superoxide anion production. The reduced CAT level in RA is due to its inactivation by H_2_O_2_ and suggests that these enzymes may play an important role in the rheumatic process and increased oxidative stress [57].

The GSH is a predominant low molecular weight thiol in the cytoplasm, which protects the tissue against in vivo toxicity of sulfhydryl—binding toxicants [58, 59]. The level of GSH appears to be reflux mechanism to protect against extracellular free radicals in chronic arthritis [60]. Glutathione is endogenously synthesized in the liver and is the first line of defence against peroxidation. Glutathione exists in the oxidized and reduced forms which are interconvertible. The reduced GSH, in turn, keeps up the cellular level of the active form of Vit-C. GSH plays an important role in the protection of cells and tissue structure [61]. Many pathological conditions are associated with decreased GSH levels. This could be due to several reasons. For instance, oxidative stress could cause GSH loss through oxidation [62].

Lipid peroxidation (LPO) is an important marker of oxidative stress and is analyzed by malonaldehyde. Increased ROS levels in RA may result in a prooxidation environment, which in turn could result in increased MDA levels.

As a result, LPO may have a role in the pathogenesis of the RA [57]. In the present study, AE and AEC significantly decreased the LPO level in CFA-induced arthritis rats probably indicating the prevention of the cell damage by reducing oxidative stress. In present study AE and AEC significantly increased the levels of SOD, CAT, and GSH possibly by preventing the inactivation of these enzymes by H_2_O_2_ or by reducing the oxidative stress.

Studies have revealed increased nitric oxide (NO) levels in the serum and synovial fluids of arthritic patients owing to the upregulation of inducible nitric oxide synthase (iNOS), indicating thereby a role of NO in arthritis. In experimental models of arthritis, selective inhibitors of iNOS have been observed to ameliorate the symptoms of joint inflammation [63]. NO levels were found to be drastically increased in disease controls indicating oxidative stress due to inflammation. The AE and AEC treatment have significantly prevented the rise in NO; the tentative mechanism maybe like treatment had prevented the formation of ROS or helped to boost the natural antioxidant system of body by preventing the disturbance in normal function.

Assessment of liver injury is generally done by ascertaining the levels of biomarkers such as SGOT, SGPT, and ALP. Elevated levels of these enzymes in serum suggest injury to the architecture of hepatic cells resulting in leaching of these enzymes into the circulation. Liver impairment is a typical feature in adjuvant arthritis. Tissue damage in adjuvant induced arthritis was assessed based on enzyme levels in serum. The present study in which significant rise in the level of aminotransferase was observed in animals treated with Freund's complete adjuvant suggests that it might be released from the damaged cells of the liver [64]. In the present study, AE and AEC treated group showed a significant improvement in serum SGOT, serum SGPT, and ALP levels, thus indicating anti-inflammatory activity which may be due to the prevention of cell damage via restoration of natural antioxidants of body.

## Supplementary Material

A growing body of evidence suggests a potential interplay between AE (Aloe emodin) and AEC (4,5-Dihydroxy-9,10-dioxo-9,10-dihydro anthracene-2-carboxylic acid). In fact, AEC have a protective role as compare to AE on inflammatory and arthritic abnormalities besides Increase specificity lowering effect, and their Anti-inflammatory potentials could be partly related to a mechanism by inhibiting COX-2 (Figure S1).

## Figures and Tables

**Table 1:** The docking score and binding free energy of aloe emodin and its derivative.

Sr. number	Compounds	Total number of conformers	Dock score	Δ*G* (total binding energy) KCal	Total number of interactions
1	AE	242	−4.7811	−616.3793	**8**
2	AEC	**26**	**−6.3658**	**−662.8458**	**27**

**Table 2:** Interactions of AE reference ligand with protein 4COX.

Amino acid	Interaction	Number of interaction	Atom of ligand
GLU 553A	Hydrogen bond	1	H
GLU 553A	Van der Waals	1	H
LYS 557A	Hydrogen bond	1	O
LYS 557A	Van der Waals	5	C, O

**Table 3:** Interactions of AEC reference ligand with protein 4COX.

Amino acid	Interaction	Number of interaction	Atom of ligand
PHE556A	Van der Waals	1	C
LYS557A	Van der Waals	8	C
ASN560A	Van der Waals	11	O, H, C
THR561A	Van der Waals	7	C, H

**Table 4:** Effect of AE and AEC on paw edema volume in carrageenan induced paw edema in rats.

Groups	Paw edema volume in mL at						
	0.5 h	1 h	2 h	3 h	4 h	5 h	24 h
I Control	0.44 ± 0.007	0.50 ± 0.01	0.54 ± 0.009	0.54 ± 0.006	0.56 ± 0.008	0.57 ± 0.009	0.27 ± 0.009
II-Std.	0.30 ± 0.008^∗∗^	0.35 ± 0.07^∗∗^	0.34 ± 0.009^∗∗^	0.32 ± 0.008^∗∗^	0.27 ± 0.008^∗∗^	0.24 ± 0.004^∗∗^	0.08 ± 0.007^∗∗^
III-AE 25	0.42 ± 0.007	0.48 ± 0.012	0.50 ± 0.006	0.52 ± 0.006	0.53 ± 0.006	0.54 ± 0.007	0.25 ± 0.004
IV-AE 50	0.40 ± 0.009^∗^	0.46 ± 0.01^∗^	0.47 ± 0.006^∗∗^	0.45 ± 0.01^∗∗^	0.41 ± 0.009^∗∗^	0.39 ± 0.008^∗∗^	0.20 ± 0.007^∗∗^
V-AE 75	0.36 ± 0.008^∗∗^	0.41 ± 0.01^∗∗^	0.42 ± 0.013^∗∗^	0.40 ± 0.010^∗∗^	0.38 ± 0.007^∗∗^	0.36 ± 0.008^∗∗^	0.17 ± 0.009^∗∗^
VI-AEC 25	0.41 ± 0.006	0.48 ± 0.009	0.49 ± 0.008	0.51 ± 0.007	0.53 ± 0.007	0.53 ± 0.008	0.25 ± 0.006
VII-AEC 50	0.35 ± 0.007^∗∗^	0.40 ± 0.007^∗∗^	0.41 ± 0.009^∗∗^	0.41 ± 0.001^∗∗^	0.38 ± 0.010^∗∗^	0.35 ± 0.007^∗∗^	0.16 ± 0.004^∗∗^
VIII-AEC 75	0.35 ± 0.011^∗∗^	0.38 ± 0.008^∗∗^	0.39 ± 0.006^∗∗^	0.37 ± 0.012^∗∗^	0.35 ± 0.025^∗∗^	0.33 ± 0.005^∗∗^	0.13 ± 0.009^∗∗^

Results are presented as mean ± SEM (*n* = 6). The data was analysed using one-way analysis of variance (ANOVA) followed by Dunnett test. ^∗^ *P* < 0.05, ^∗∗^ *P* < 0.01 when compared with arthritic control group. AC: arthritic control; Std.: Diclofenac sodium 10 mg/kg p.o.; AE: aloe emodin (25, 50, and 75 mg/kg p.o.); AEC: carboxylic acid derivative of aloe emodin (25, 50, and 75 mg/kg p.o.).

**Table 5:** Effect of AE and AEC on hematological parameters (RBCs, WBCs, and Hb) in Freund's complete adjuvant induced arthritic rats.

Groups (*n* = 6)	RBC (millions/cubic mm)	WBC (10^3^/cubic mm)	Hb (gm%)
I-AC	5.83 ± 0.600	14.83 ± 0.872	9.16 ± 0.477
II-Std.	9.66 ± 0.666^∗∗^	6.83 ± 0.703^∗∗^	14.83 ± 0.872^∗∗^
III-AE 25	6.50 ± 0.562	12.33 ± 1.054	10.50 ± 0.763
IV-AE 50	8.66 ± 0.333^∗∗^	9.16 ± 0.477^∗∗^	12.50 ± 0.428^∗∗^
V-AE 75	8.83 ± 0.703^∗∗^	8.50 ± 0.428^∗∗^	12.33 ± 0.557^∗∗^
VI-AEC 25	6.33 ± 0.494	11.83 ± 0.792^∗^	11.00 ± 0.577
VII-AEC 50	8.83 ± 0.401^∗∗^	8.83 ± 0.477^∗∗^	12.83 ± 0.477^∗∗^
VIII-AEC 75	9.00 ± 0.577^∗∗^	8.00 ± 0.365^∗∗^	13.16 ± 0.703^∗∗^

Results are presented as mean ± SEM (*n* = 6). The data was analysed using one-way analysis of variance (ANOVA) followed by Dunnett test. ^∗^ *P* < 0.05, ^∗∗^ *P* < 0.01 when compared with arthritic control group. AC: arthritic control; Std.: Diclofenac sodium 10 mg/kg p.o.; AE: aloe emodin (25, 50, and 75 mg/kg p.o.); AEC: carboxylic acid derivative of aloe emodin (25, 50, and 75 mg/kg p.o.).

**Table 6:** Effect of AE and AEC on RF and ESR levels in Freund's complete adjuvant induced arthritic rats.

Groups (*n* = 6)	RF (IU/L)	ESR (mm/hr)
I-AC	46.16 ± 1.138	10.83 ± 0.600
II-Std.	25.66 ± 0.988^∗∗^	5.50 ± 0.670^∗∗^
III-AE 25	42.83 ± 0.792	9.33 ± 0.494
IV-AE 50	36.83 ± 0.909^∗∗^	7.50 ± 0.428^∗∗^
V-AE 75	34.16 ± 0.600^∗∗^	6.83 ± 0.600^∗∗^
VI-AEC 25	41.83 ± 0.872^∗^	9.00 ± 0.516
VII-AEC 50	32.50 ± 0.846^∗∗^	7.00 ± 0.365^∗∗^
VIII-AEC 75	29.33 ± 1.054^∗∗^	6.16 ± 0.477^∗∗^

Results are presented as mean ± SEM (*n* = 6). The data was analysed using one-way analysis of variance (ANOVA) followed by Dunnett test. ^∗^ *P* < 0.05, ^∗∗^ *P* < 0.01 when compared with arthritic control group. AC: arthritic control; Std.: Diclofenac sodium 10 mg/kg p.o.; AE: aloe emodin (25, 50, and 75 mg/kg p.o.); AEC: carboxylic acid derivative of aloe emodin (25, 50, and 75 mg/kg p.o.).

**Table 7:** Effect of AE and AEC on antioxidant levels in Freund's complete adjuvant induced arthritic rats.

Group (*n* = 6)	LPO (nM of MDA/g of wet tissue)	GSH (*μ*g of GSH/g of wet tissue)	CAT (*µ*M of H_2_O_2_/g of wet tissue/minute)	SOD (units/mg of wet tissue)	NO (*μ*M/g of wet tissue)
I-AC	27.16 ± 0.945	17.16 ± 1.138	4.81 ± 0.219	22.20 ± 0.998	50.3 ± 0.905
II-Std.	15.66 ± 0.881^∗∗^	22.16 ± 0.833^∗∗^	5.03 ± 0.197^∗∗^	49.71 ± 0.786^∗∗^	25.17 ± 0.525^∗∗^
III-AE 25	24.33 ± 0.7601	19.83 ± 0.872	5.11 ± 0.192	23.89 ± 1.042	47.53 ± 0.496
IV-AE 50	20.33 ± 0.614^∗∗^	22.16 ± 0.792^∗∗^	6.18 ± 0.149^∗∗^	35.37 ± 0.765^∗∗^	40.37 ± 0.632^∗∗^
V-AE 75	18.50 ± 1.176^∗∗^	23.16 ± 0.872^∗∗^	6.73 ± 0.135^∗∗^	41.70 ± 1.034^∗∗^	35.03 ± 0.509^∗∗^
VI-AEC 25	23.16 ± 0.833^∗^	20.66 ± 0.614^∗^	5.48 ± 0.130^∗^	26.23 ± 0.656^∗^	47.14 ± 0.521^∗^
VII-AEC 50	20.16 ± 0.945^∗∗^	23.00 ± 0.577^∗∗^	6.26 ± 0.114^∗∗^	40.35 ± 0.939^∗∗^	38.58 ± 0.852^∗∗^
VIII-AEC 75	17.33 ± 0.918^∗∗^	24.50 ± 0.763^∗∗^	7.05 ± 0.102^∗∗^	43.57 ± 0.635^∗∗^	33.07 ± 0.948^∗∗^

Results are presented as mean ± SEM (*n* = 6). The data was analysed using one-way analysis of variance (ANOVA) followed by Dunnett test. ^∗^ *P* < 0.05, ^∗∗^ *P* < 0.01 when compared with arthritic control group. AC: arthritic control; Std.: Diclofenac sodium 10 mg/kg p.o.; AE: aloe emodin (25, 50, and 75 mg/kg p.o.); AEC: carboxylic acid derivative of aloe emodin (25, 50, and 75 mg/kg p.o.).
