# Supplementary material for: Anti-Inflammatory and Antiarthritic Activity of Anthraquinone Derivatives in Rodents
Source: Int J Inflam. 2014 Dec 24;2014:690596. doi: 10.1155/2014/690596 (PMC4290027; doi:10.1155/2014/690596)
Supplement: Supplementary file 1 — A growing body of evidence suggests a potential interplay between AE (Aloe emodin) and AEC (4,5-Dihydroxy-9,10-dioxo-9,10-dihydro anthracene-2-carboxylic acid). In fact, AEC have a protective role as compare to AE on inflammatory and arthritic abnormalities besides Increase specificity lowering effect, and their Anti-inflammatory potentials could be partly related to a mechanism by inhibiting COX-2 (Figure S1). [file 690596.f1.zip › mat.690596.v2.pdf]

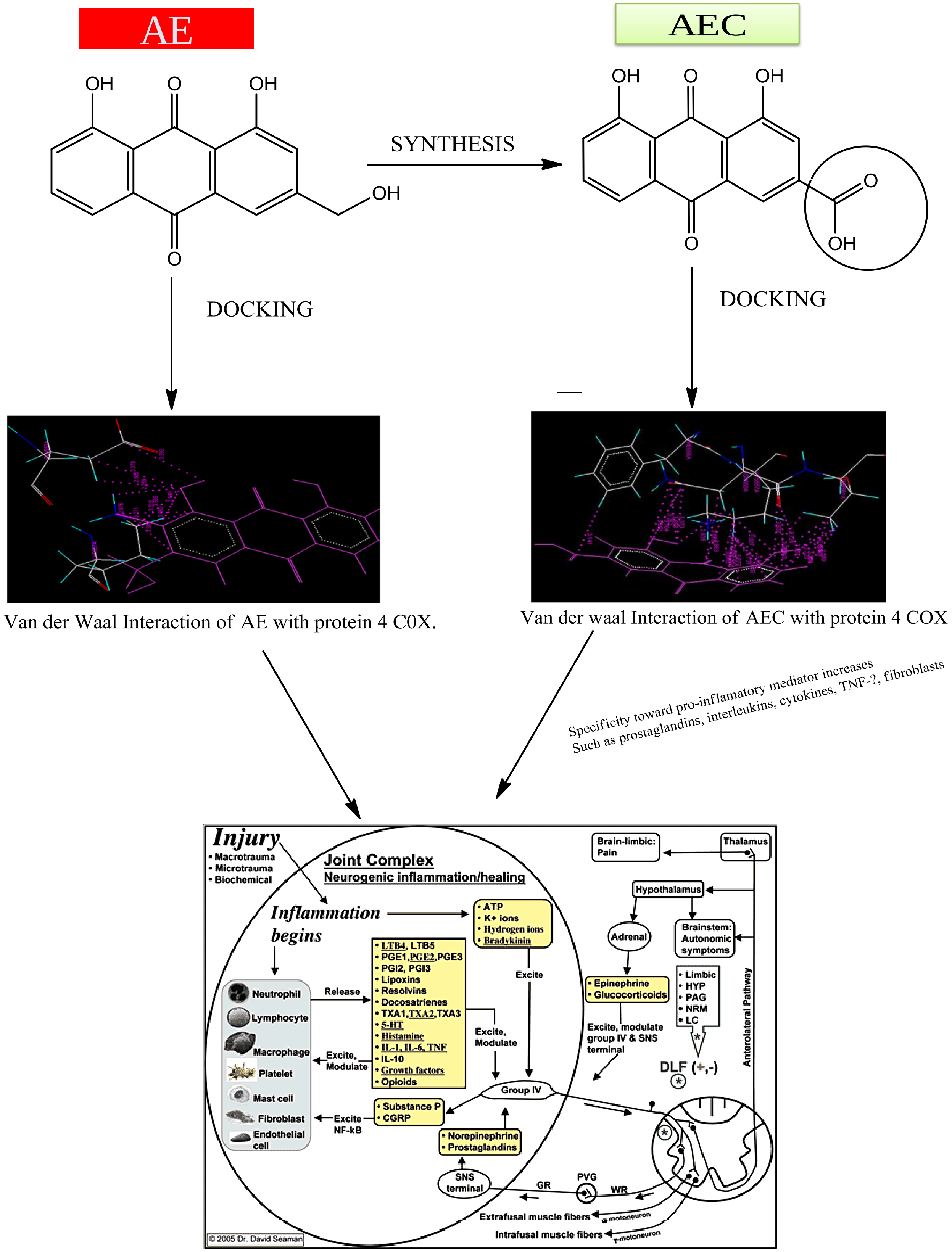

AE
AEC
OH
O
OH
OH
O
SYNTHESIS
OH
O
OH
O
O
OH
DOCKING
DOCKING
Van der Waal Interaction of AE with protein 4 C0X.
Van der waal Interaction of AEC with protein 4 COX
Specificity toward pro-inflamatory mediator increases
Such as prostaglandins, interleukins, cytokines, TNF-?, fibroblasts
Injury
• Macrotrauma
• Microtrauma
• Biochemical
Joint Complex
Neurogenic inflammation/healing
Inflammation begins
• ATP
• K+ ions
• Hydrogen ions
• Bradykinin
Excite
• LTB4, LTB5
• PGE1,PGE2,PGE3
• PGI2, PGI3
• Lipoxins
• Resolvins
• Docosatrienes
• TXA1,TXA2,TXA3
• 5-HT
• Histamine
• IL-1, IL-6, TNF
• IL-10
• Growth factors
• Opioids
Release
Excite, Modulate
Excite, Modulate
Neutrophil
Lymphocyte
Macrophage
Platelet
Mast cell
Fibroblast
Endothelial cell
Group IV
• Substance P
• CGRP
Excite NF-kB
• Norepinephrine
• Prostaglandins
SNS terminal
GR
PVG
WR
Extrafusal muscle fibers
α-motoneuron
Intrafusal muscle fibers
γ-motoneuron
Brain-limbic: Pain
Thalamus
Hypothalamus
Adrenal
Brainstem: Autonomic symptoms
• Epinephrine
• Glucocorticoids
Excite, modulate group IV & SNS terminal
• Limbic
• HYP
• PAG
• NRM
• LC
DLF (+,-)
Anterolateral Pathway
© 2005 Dr. David Seaman
