# Supplementary figures and images for: Anti-Inflammatory and Antiarthritic Activity of Anthraquinone Derivatives in Rodents
Source: Int J Inflam. 2014 Dec 24;2014:690596. doi: 10.1155/2014/690596 (PMC4290027; doi:10.1155/2014/690596)

Figure S1:

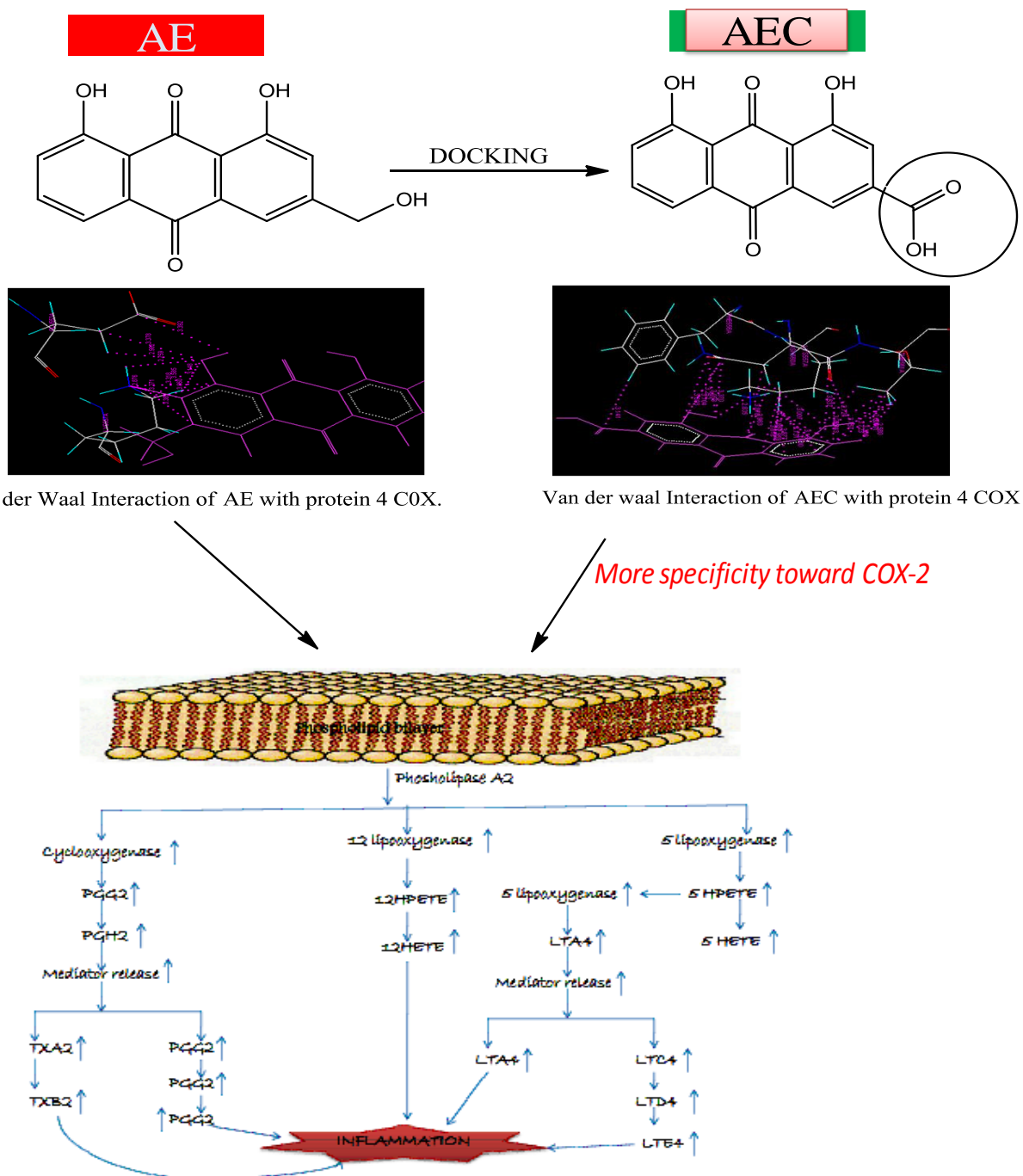

Supplement: Supplementary file 1 — A growing body of evidence suggests a potential interplay between AE (Aloe emodin) and AEC (4,5-Dihydroxy-9,10-dioxo-9,10-dihydro anthracene-2-carboxylic acid). In fact, AEC have a protective role as compare to AE on inflammatory and arthritic abnormalities besides Increase specificity lowering effect, and their Anti-inflammatory potentials could be partly related to a mechanism by inhibiting COX-2 (Figure S1). [file 690596.f1.zip › Figure S1.pdf]
